# Spanish Readers Skip Articles Regardless of Gender and Number Agreement

**DOI:** 10.3390/jemr19010006

**Published:** 2026-01-09

**Authors:** Marina Serrano-Carot, Bernhard Angele

**Affiliations:** Faculty of Education and Language, Universidad Antonio de Nebrija, Calle de Asura, 90, 28043 Madrid, Spain

**Keywords:** parafoveal processing, eye movements, grammatical agreement, boundary paradigm, reading comprehension, Spanish

## Abstract

Articles are among the most frequently encountered words during reading; however, it is not clear how deeply they are usually processed. This study examines whether native Spanish speakers use parafoveal article–noun agreement information to guide eye movements during reading. Using the gaze-contingent boundary paradigm, we manipulated the parafoveal preview of articles across two experiments. In Experiment 1, we manipulated gender agreement between the previews readers received of definite articles and the subsequent nouns (e.g., *la mesa* vs. el* mesa). In Experiment 2, we manipulated grammatical gender and number agreement between parafoveal article previews and the subsequent nouns jointly (e.g., los* mesa vs. *una mesa*). We found no evidence that parafoveal article–noun gender or number agreement affected article skipping probability, suggesting that initial parafoveal processing of articles does not extend to their grammatical properties. However, we observed increased total viewing time on the noun following mismatching previews, suggesting that, while the decision of whether to skip an article is taken largely without considering the grammatical properties of the upcoming words, readers do need more time to recover from the grammatical mismatch afterwards. We discuss the results in the context of current models of eye-movement control during reading.

## 1. Introduction

Reading is a complex cognitive task that involves the integration of visual and linguistic information. As readers move their gaze across text, they not only process the words they are currently fixating on (foveal processing) but also gather information from words that are not yet fixated (parafoveal processing). Efficient reading involves integrating this parafoveal information to guide upcoming eye movements and facilitate fluent comprehension, but it is still debated to what extent different types of parafoveal information influence both eye movements and ongoing language processing [1].

Importantly, both linguistic and visual factors contribute to word skipping behavior [2]. Word skipping is driven by an interplay between oculomotor constraints and linguistic processing. While word length and launch site are primary determinants of skipping behavior, readers can also integrate parafoveal information with sentence context to guide their gaze. However, the extent to which complex syntactic information, such as gender agreement, is processed parafoveally remains a key debate in models of eye-movement control. Word frequency has a major effect on both fixation duration and skipping probability [3,4] but other properties such as predictability and word length also play a role. Word length in particular can affect skipping probability both in terms of word identification—shorter words may be easier to identify as fewer letters need to be recognized—and due to the properties of the oculomotor system: it is harder to target short words and to make very short saccades. Because of this, the launch site of a saccade also plays a role in whether a word is skipped or not, with saccades launched close to a word being more likely to result in skipping it. These findings highlight that skipping reflects an interplay between cognitive and oculomotor processes, rather than being driven solely by linguistic factors.

In Spanish, definite and indefinite articles are skipped very often, as they occur extremely frequently in the Spanish language: *la* (definite, singular, feminine) has 42,548 occurrences per million according to the EsPal lexical database [5], while *el* (definite, singular, masculine) has 31,372 occurrences, followed by the plural definite articles *los* (17,238) and *las* (10,959) and the singular indefinite articles *un* (10,388) and *una* (8687). Although there are substantial numerical differences among these frequency values, such variation is unlikely to translate into differences in skipping probability or fixation durations, since word frequency effects are known to be most pronounced for low- to medium-frequency words (i.e., below 100 occurrences per million, [6]). Consequently, a ceiling effect is expected at such extreme frequencies: all articles, even the relatively less frequent ones, are likely processed with comparable efficiency due to their overall ubiquity in the lexicon. Processing of all short, high-frequency function words may be equally highly automatized and extremely efficient.

The ability to skip short and easy-to-identify words, that is, to process them completely parafoveally without having to fixate them, is particularly important in order to read quickly and efficiently [2]. Previous research suggests that, when making the decision of whether to fixate or skip an upcoming word, readers can integrate parafoveal information with information about the sentence context [7]. However, there is evidence that they do not always seem to take the sentence context into account: Using the gaze-contingent display change paradigm [8], Angele and Rayner [9] manipulated the parafoveal preview readers received for a three-letter target content word embedded in a sentence (e.g., “far” in “The house was very far from the road.”) while they were fixating to the left of the target word (e.g., on the pre-boundary word “very”). Once a participant’s gaze crossed the invisible boundary placed to the left of the target word, a display change was triggered that replaced the preview with the actual target word. Angele and Rayner found that readers were more likely to skip the target word when the preview for the target word had been *the*, which was always grammatically invalid given the sentence context (e.g., *the* cannot follow “The house was very”), compared to the identical control preview condition (e.g., *far*). This was replicated for previews that were high-frequency target words (e.g., *all* as a preview for *ant* in the sentence “There was a massive ant infestation in the house” [10] and even in cases where there was extremely high contextual constraint, e.g., when *the* was presented as a preview for *die* in the sentence “If you are shot in the heart, you will surely die immediately” [11]. Angele and Rayner [9] suggested that very short and highly frequent function words (such as articles) are skipped automatically, without integrating the grammatical information from the preceding sentence context.

Recent research on function word processing further complicates this picture. Staub et al. [12] showed that readers often fail to notice omissions or repetitions of short function words such as *of* or *to*, and that when such errors go unnoticed, they produce no detectable disruption in eye-movement patterns. These findings challenge the assumption that all words are incrementally processed and integrated into syntactic structure and instead suggest that readers may engage in rapid *perceptual inference* processes that “correct” noisy linguistic input to preserve fluent comprehension. Within the noisy-channel framework of sentence comprehension [13], this means that readers infer the intended message even when the visual input contains inconsistencies. Applied to parafoveal processing, this perspective raises the possibility that apparent insensitivity to grammatical mismatches—such as article–noun agreement violations—might reflect not the absence of processing, but successful perceptual correction of the mismatch before it reaches conscious awareness or influences saccade programming.

The lack of an effect of the sentence context may be due to issues with retrieving previously accessed words in time to influence the skipping decision. A related but different situation arises when the article and the incompatible context are both in the parafovea. In this case, retrieval of previous words from working memory should not play a role. For a reader to detect such a conflict parafoveally, both the article and the subsequent noun must be processed to a sufficient degree before fixation to retrieve their grammatical features and identify the mismatch. Crucially, this mismatch information must then be accessible to the oculomotor system in time to influence the next saccade target. On the other hand, it is also plausible that parafoveal information is processed jointly across multiple words, and that a salient mismatch, such as one in gender or number, can interrupt ongoing processing or trigger attention shifts that increase fixation probability on the article. This idea was first put forward by Radach [14] as the *Word Grouping Hypothesis.* On the other hand, if short, high-frequency words such as articles are processed completely automatically and isolated from any contextual information no matter whether this information precedes or follows the article, a parafoveal agreement violation should have no effect at all on processing.

It is easy to design a manipulation creating a situation where two parafoveal words contain conflicting information in a language with a rich morphosyntactic system, such as Spanish, where nearly every noun carries grammatical gender and often number marking. For example, the noun *mesa* (table) is feminine (and marked as such by the ending -*a*) and must be accompanied by a feminine article, e.g., *la mesa* (the table) or *una mesa* (a table). Similarly, the plural *mesas* needs to be accompanied by a plural feminine article (*las mesas* or *unas mesas*). In such languages, mismatches like “el* mesa” or “los* mesas” violate article–noun gender and/or number agreement. When presented auditorily or foveally, such violations are usually very salient to native speakers and have clear effects on processing [15,16,17].

For a reader to detect such an agreement violation parafoveally, both the article and the subsequent noun must be processed to a sufficient degree before fixation to retrieve their grammatical features and identify the mismatch. Crucially, this mismatch information must then be accessible to the oculomotor system in time to influence the next saccade target. On the other hand, it is also plausible that parafoveal information is processed jointly across multiple words, and that a salient mismatch, such as one in gender or number, can interrupt ongoing processing or trigger attention shifts that increase fixation probability on the article. This idea was first put forward by Radach [14] as the *Word Grouping Hypothesis.* Of course, if short, high-frequency words are processed completely automatically and isolated from any contextual information, a parafoveal agreement violation should have no effect at all on processing.

If readers can detect grammatical mismatches in the parafovea, this directly informs the debate on serial versus parallel processing in reading [1,18,19] as detecting a syntactic violation requires the processing of two parafoveal words either at the same time or in very quick succession.

Within the serial framework, the E-Z Reader model [7,20] assumes that attention shifts sequentially from word to word, and that skipping occurs when a word has been at least partially lexically processed parafoveally, triggering a saccade to the next word. This model predicts that skipping is sensitive to lexical factors such as frequency and predictability, and that parafoveal processing—and, consequently, skipping—is reduced when the currently fixated word imposes a higher foveal load.

In contrast, attention-gradient models such as SWIFT [21] and Glenmore [22] assume that multiple words are processed in parallel, with attention distributed across a gradient spanning several words. Skipping, in these accounts, emerges from the interaction between lexical activation and oculomotor constraints.

Importantly, none of the models discussed so far make any claim about syntactic effects, particularly effects of grammatical agreement, on skipping probability and fixation durations. Such effects may emerge from models such as the Rational Reading Model [23,24] a Bayesian approach in which uncertainty about word identities can influence saccade targeting both before and after words have been fixated for the first time, and SEAM (Sentence-Processing and Eye-Movement Activation-Coupled Model, [25]) a model combining Lewis and Vasishth’s [26] model that assumes sentence reading times are determined by memory-retrieval processes and the attention-gradient and oculomotor components of SWIFT.

If readers can detect grammatical mismatches parafoveally, it would imply that syntactic information from multiple words is either accessed in parallel or the two parafoveal words are identified serially in very quick succession, as gender information from the article would need to be integrated with the noun. As such, a finding that readers can detect parafoveal syntactic violations and adapt their eye movement behavior immediately would severely constrain serial models but be largely compatible with parallel models. Conversely, the absence of such effects would be compatible with models that assume serial processing, at least at the syntactic level, or parallel accounts that assume that syntactic information such as gender is either not integrated at all during parafoveal processing or is integrated so late that it does not influence eye movements.

The only study so far that has tested this directly was performed by Schwalm and Radach [27], who found that German readers, when presented with previews of an article featuring a gender agreement violation (e.g., *die*, the nominative/accusative singular feminine article preceding *Fisch*, a masculine singular noun) articles with such gender agreement previews were fixated more often than articles with correct agreement previews (*den Fisch,* with *den* being the accusative singular masculine article). However, in a follow-up experiment where the preview manipulation occurred on the noun rather than the article (e.g., *der Fisch* vs. *der Katze*, with *Katze* being a singular feminine noun), they failed to replicate this effect on skipping of the article, although later measures revealed increased disruption downstream. Complicating the interpretation of Schwalm and Radach’s results is the German case system: *der Fisch* is nominative masculine singular, but *der Katze* is not necessarily incorrect, as it can be dative feminine singular. In some of the sentences Schwalm and Radach used, interpreting the noun as dative leads to a potentially valid sentence continuation which is only disambiguated further downstream in the sentence. There are similar issues with other articles. For example, *die* is not only the nominative singular feminine article, but also the universal nominative/accusative plural article, resulting in some of the nouns in the gender agreement violation condition being near neighbors of words that would fit in the sentence and not involve a syntactic mismatch As an example, in the example sentence given for Schwalm and Radach’s Experiment 1, “Der geduldige Vater zieht…” (the patient father is pulling), *die Fisch* (the fish, singular) would not be a valid continuation, but *die Fische* (the fish, plural) would be. Similarly, in the example sentence for Experiment 2, “Ein heftiger Windstoß trägt…” (A violent gust of wind is carrying…) *die Vogel* (the bird, singular) would not be a valid continuation, but *die Vögel* (the birds, plural), would be. There are many such examples in the materials of both experiments due to the ambiguity of most German articles. This may have led to a situation where the supposed gender agreement violation previews were either valid continuations of the sentence or similar enough to valid continuations to not provoke a fixation on the target article, with the syntactic or semantic violation only becoming apparent later, perhaps after a process of perceptual interference as proposed by Staub et al. [12]. In any case, the fact that the preview effect on skipping articles is not replicated in Experiment 2 means that it remains unclear whether parafoveal information from more than one word can inform skipping decisions.

Spanish does not express case in either articles or nouns, and all articles are unambiguous. The only exception is the rule that feminine, singular words that begin with “a” and are stressed on the first syllable use “el” and “un” rather than “la” or “una” (e.g., “el agua”, but “las aguas”). Even so, there is no ambiguity, as, in those words, “la” can never be used (e.g., “la agua” is categorically incorrect). We did not include such words in the present study, Because of this, the ambiguity issue does not present itself. Additionally, the Spanish singular definite articles *el* and *la* are only two characters long, which means that the subsequent noun is closer to the center of vision and likely to receive more processing than in languages with three-letter articles. An additional benefit is that, unlike in most other romance languages, the singular definite masculine article *el* has a distinctive word shape (inline character + ascender) compared to the feminine article *la* (ascender + inline character), while the plural definite articles *los* and *las* have very similar word shapes. It is important to note that word length is a primary determinant of skipping behavior. As Drieghe et al. [2] demonstrated, word length can be a decisive factor, showing that an unexpected two-letter word is skipped more often than an expected four-letter word. In line with the E-OVP model [28], this suggests that for very short and frequent words like Spanish articles, the decision to skip may be driven more by their physical length and visual characteristics than by a detailed linguistic analysis of their agreement with the following noun.

We conducted two eye-tracking experiments in Spanish using the gaze-contingent boundary paradigm [8] in order to examine whether parafoveal processing is sensitive to article–noun agreement. In doing so, we not only manipulated grammatical congruence (analogous to Schwalm and Radach’s Experiment 1), but, due to the properties of the Spanish definite articles, Experiment 1 also was a test of whether the shape of the parafoveal preview plays a role in determining whether a parafoveal word is skipped or not by comparing the effect for singular articles to plural articles *el/la* vs. *los/las*). In Experiment 2, we manipulated the definiteness and the number of the previews in addition to their gender by showing *una* (the indefinite singular feminine article) as a preview for *los* (the definite plural masculine article) and vice versa.

## 2. Hypotheses

If readers rely exclusively on the parafoveal properties of the immediate upcoming word (e.g., the article itself) to make a skipping decision (without integrating information about the subsequent noun) then no difference is expected in article skipping rates between the identical preview condition and the dissimilar preview condition involving an agreement violation. This would suggest that readers do not process article–noun agreement prior to making a skipping decision and, more generally, that articles are processed in isolation and without taking the surrounding context into account.

Alternatively, if readers integrate article-noun agreement parafoveally and calculate their agreement, we expect that a dissimilar preview in which the article does not agree in gender and/or number with the upcoming noun would increase the probability of fixating the article compared to the identical condition. Based on previous findings for short function words, we expect the disruption to manifest primarily in later processing measures rather than in initial skipping decisions. This would reflect the reader’s sensitivity to grammatical violations in the parafovea and would imply that article–noun agreement is evaluated at least to some degree before skipping decisions are made. Of course, it is also possible that readers detect the grammatical violation but attempt to resolve it by fixating the noun rather than the article, resulting in longer fixation times on the noun and no change in article skipping.

Additionally, the agreement violation may be easier to detect for the singular articles, which are visually very different (*el* vs. *la*) compared to the plural articles, which are much more similar (*los* vs. *las*).

There may also be differences in terms of the correct gender of the article and noun. In Spanish, the masculine gender is often interpreted as the default [29]. A disruption caused by a dissimilar preview involving an agreement violation may be stronger for feminine articles and nouns as it may be harder to override the masculine default once it is established by a dissimilar preview.

In Experiment 2, where gender, number, and definiteness features are manipulated (e.g., *una mesa* vs. los* mesa), we may observe different effects than in Experiment 1, where only gender was manipulated.

Of course, the skipping decisions are only one possible impact of the preview manipulation. Just as Angele and Rayner [9], we expect to observe a preview benefit effect on the article in those cases where it is fixated. Specifically, when the preview is incorrect and no useful parafoveal information about the article was available during the previous fixations, early fixation measures such as first fixation duration and gaze duration (when the article is fixated) should be longer than in the correct preview condition, where useful information about the article could be extracted during parafoveal processing.

Finally, if the preview information is processed at all, a dissimilar preview is likely to disrupt later processing stages, leading to increased cognitive load and reanalysis demands. This is similar to what was observed in as in Schwalm and Radach’s [27] Experiment 2. If there is later disruption due to the preview manipulation, we expect longer go-past times and total viewing times compared to the identical condition, both on the article (when fixated) and on the subsequent noun. These late effects would indicate that even if an article is skipped, the system still detects and resolves the mismatch during or the processing of the noun.

## 3. Experiment 1

In Experiment 1, we manipulated the preview readers received of an article. The preview article either corresponded to the grammatical gender of the subsequent noun (e.g., *la mesa*) or it did not (el* mesa). In this experiment, only gender agreement was manipulated.

### 3.1. Method

#### 3.1.1. Participants

This study included 24 participants aged between 18 and 35 years, wo were recruited from the Nebrija University community (mean age = 23.57 years). The sample consisted of 15 women and nine men who participated in this study in exchange for a small compensation for their time (12€ for approximately one hour of participation). All participants were native Spanish speakers, reported normal or corrected-to-normal vision and no previous diagnosis of reading disorders and were naïve as to the purpose of the study. All participants gave informed consent before the experiment. The research followed the principles and guidelines of the Declaration of Helsinki, and we obtained ethical approval from the Nebrija University Research Ethics Committee (Ref. UNNE-2023-0031).

#### 3.1.2. Apparatus

An EyeLink Portable Duo eye tracker (SR Research, Ltd., Missisauga, ON, Canada) was used to record subjects’ eye movements with a sampling rate of 1000 Hz. Subjects read sentences displayed on a computer screen (Lenovo Legion Y25-30, 24.5”, Lenovo, Beijing, China) with a refresh rate of 240 Hz, ensuring very fast gaze-contingent display changes. Stimuli were presented using a computer running the OpenSesame (Version 4.0.13; [30]) with the PyGaze plugin [31] on Windows 10.

Viewing and recording were binocular, but, in line with the majority of eye tracking studies during reading, we analyzed only data from the right eye. Viewing distance was approximately 60 cm, with 3.8 letters equaling one degree of visual angle.

#### 3.1.3. Materials

The experimental materials were constructed using the EsPal linguistic database [18] from which word lists were selected based on lexical frequency (greater than 10 occurrences per million). A total of 210 high-frequency Spanish nouns were selected (105 feminine and 105 masculine) carefully matched for both lexical frequency and word length. Since 105 is not divisible by four, the counterbalancing across the four sub-conditions (e.g., masculine singular identical vs. masculine singular dissimilar) was kept as even as possible, with a maximum difference of only one item between cells across the entire experiment design. Singular nouns ranged from 4 to 6 characters, while plural forms ranged from 5 to 7 characters, accounting for the addition of “*-s*” in Spanish pluralization.

Each noun was embedded into a sentence containing a mid-sentence article-noun phrase, with the article serving as the target word. The definite articles *el*, *la*, *los*, and *las* were used, corresponding to the masculine/feminine and singular/plural forms of the definite article in Spanish. These articles were chosen because they function as the closest Spanish equivalents to the English definite article *the*, thus offering a relevant point of comparison for studies on article processing.

The experiment comprised 210 Spanish sentences, of these, 105 sentences included a masculine noun (e.g., *coche*) and 105 included a feminine noun (e.g., *mesa*). Each participant read a total of 220 sentences, including 10 practice sentences at the beginning of the experiment. Additionally, we created comprehension questions for a third of the sentences. For each sentence, two grammatical number conditions were created: one with the noun in singular form (*coche*/*mesa*), and one in plural form (*coches*/*mesas*). This contributed to a balanced design, with equal representation of gender (masculine/feminine) and number (singular/plural), resulting in four experimental conditions: masculine singular, masculine plural, feminine singular, and feminine plural. Additionally, following the gaze contingent paradigm each sentence we had two versions based on article-noun agreement: an identical version, in which the article and noun agreed in gender and number, resulting in a grammatically correct phrase (e.g., *el coche*, *las mesas*), and a dissimilar version, in which the article and noun mismatched either in gender or number (e.g., la* coche, los* mesa), thus creating a syntactic anomaly. These versions allowed us to manipulate grammatical congruence systematically and examine the effects of article-noun agreement on reading behavior. The distribution of items across conditions is presented in Table 1. Appendix A.1 contains all experimental sentences used in Experiment 1.

#### 3.1.4. Design

The experiment followed a within-subjects design, in which all participants were exposed to all experimental conditions. The independent variables included preview type (identical vs. dissimilar), grammatical number (singular vs. plural), and grammatical gender (masculine vs. feminine). Preview type and number were manipulated within subjects and items, whereas gender was manipulated within subjects but across different sets of items.

To avoid fatigue, the 210 sentences were divided into three blocks of 70 sentences and were presented separately, with short task breaks between them. The order in which the blocks were presented was counterbalanced across participants: one-third of the participants saw the blocks in the order 1-2-3, another third in the order 2-3-1, and the remaining third in the order 3-1-2.

The experiment included two versions of the stimuli: Each version contained half of the 210 experimental sentences in singular form and the other half in plural form, while the remaining sentences appeared in the complementary version. This ensured that each participant saw only one version of each sentence, and that all items were tested equally across conditions.

The preview manipulation was implemented using the gaze-contingent boundary paradigm. In the identical condition, the previewed word was visually identical to the word that appeared after the boundary, meaning that although a display change technically occurred, it was imperceptible to the participant. In contrast, the dissimilar condition introduced a grammatical mismatch between the article and the noun (e.g., las* coche), with an invisible boundary placed immediately after the word preceding the article-noun phrase. When the participant’s gaze crossed this boundary, the article was replaced by the grammatically correct one (e.g., *el coche*), resulting in a visible display change that was nevertheless nearly undetectable by the participant due to saccadic suppression.

All conditions were fully counterbalanced across participants with respect to block order, noun number (singular/plural), grammatical gender (masculine/feminine), and preview condition (identical/dissimilar). This counterbalancing also ensured that each item appeared equally often in each condition across the sample, and that potential effects of fatigue, order, or item-specific characteristics were minimized.

#### 3.1.5. Procedure

The 210 sentences were presented using the OpenSesame software (Version 4.0.13; [30]). Participants initiated each sentence presentation by fixating on a rectangular gaze target located on the left side of the screen for more than 250 ms. Following this fixation, the sentence appeared, with the first word displayed at the location of the gaze target. An invisible boundary was placed at the end of the word preceding the target article. While participants fixated to the left of this boundary, the target article was replaced by either an identical preview (i.e., the same article) or a dissimilar preview (i.e., the opposite-gender form of the article). Once the participant’s right-eye gaze position crossed to the right of the boundary, the display updated to show the actual target article, regardless of the preview condition (in the identical preview condition, this update did not change any of the pixels on the screen and was therefore invisible).

Participants concluded each trial by fixating on a small square fixation target located in the lower right corner of the screen. A two-alternative forced-choice (yes/no) comprehension question was presented for 24 sentences in each block. Responses were recorded using a Black Box Toolkit (BBTK) five-button USB response pad (Black Box Toolkit, Ltd., Sheffield, UK). Once the comprehension question was answered, the trial ended, and the next sentence was presented, continuing until the end of the block.

After completing the experiment, participants were asked whether they had noticed any display changes during the task. Data from participants who reported having seen more than ten changes were excluded from the analysis, as such cases were typically due to calibration issues that could compromise within-subject comparability. Noticing display changes was rare: the average number reported was 3, and most participants simply mentioned seeing a slight flicker rather than a noticeable change. Three additional participants who reported seeing more than ten changes were removed from the analysis.

#### 3.1.6. Data Analysis

The data were analyzed to examine the effects of article-noun agreement on reading behavior, with a particular focus on grammatical congruency, gender, and number. The main dependent variables included skipping probability, first fixation duration (FFD, the duration of the first fixation on the word), gaze duration (GD, the duration of the first fixation plus all refixations prior to leaving the word region), go-past time (i.e., regression path duration, the time from entering the word region for the first time from the left to leaving it towards the right, including all regressions to previous words in the sentence) and total viewing time (TVT, the sum of all fixations on the word region including both first pass and subsequent regressions) for the pre-boundary region (the word to the left of the boundary), the target article and post-target word regions.

Analyses were conducted using Bayesian linear mixed-effects modeling approaches in R. The Bayesian models were fitted using the “brms” package [32]. Skipping probabilities were modelled using the Bernoulli distribution, while log-transformed fixation times were modelled as Gaussian distributions. We used weakly informative priors (a Gaussian distribution with a mean of 0 and an SD of 200) for the regression coefficients, and the default priors set by *brms* for all other parameters. Each model was fitted using four chains with 5000 iterations each, for which 1000 were warm-up iterations. The models converged successfully (all R^s = 1.00). We report the mean, the estimates (b) and the 95% Bayesian credible intervals (95% CI) based on the posterior distribution of each parameter. When reporting the results, we will describe the evidence for an effect as credible when 0 is not a credible value for the corresponding coefficient (i.e., if it is not part of the 95% CI). However, it is important to note that this is not a formal hypothesis test as they are used in frequentist statistics but merely an aid in describing and interpreting the results.

All models incorporated three fixed effects—preview condition, grammatical gender, and grammatical number—as well as their interactions. The preview condition (identical vs. dissimilar) was contrast-coded as –0.5 for identical and +0.5 for dissimilar. A positive coefficient thus indicated higher fixation probability in the dissimilar condition, suggesting greater disruption due to article-noun mismatches. Grammatical gender was contrast-coded as –0.5 for feminine and +0.5 for masculine, allowing a positive coefficient to indicate higher fixation probability for masculine nouns. Grammatical number was coded as –0.5 for singular and +0.5 for plural, such that a positive coefficient would reflect higher fixation probability for plural forms (according to their higher word length). In addition to the fixed effects, all models included the maximal random effects structure as recommended by Barr et al. [33] incorporating random intercepts and random slopes for all fixed effects over both subjects and items.

Data exclusion criteria were applied to ensure the integrity of the analysis. Trials were removed if they involved track losses, display changes occurring more than 10 ms after fixation onset, or blinks occurring immediately before or during fixation on the target article. Fixations shorter than 80 ms and located within one character space of another fixation were either merged or excluded, and fixations exceeding 800 ms were also discarded. For aggregate fixation measures, gaze durations exceeding 1500 ms and go-past or total viewing times exceeding 4000 ms were excluded, using intentionally conservative thresholds to preserve valid data.

At the participant level, data were excluded if a participant answered fewer than 85% of comprehension questions correctly or if fewer than 25% of their trials met the inclusion criteria after preprocessing. Additionally, participants who reported perceiving more than 10 display changes were removed from the sample to ensure comparability across participants. In such cases, new participants were recruited to maintain balance across experimental conditions. All preprocessing steps were implemented with custom R scripts based on data files created with the SR Research EDF2ASC tool.

### 3.2. Results and Discussion

After preprocessing, a 4.43% of the trials were excluded based on the criteria described above. Specifically, 2.98% were too long fixations (>800 ms), 0.67% were removed due to proximity to blinks, and a 0.78% due to short fixations (<80 ms). Table 2 shows mean fixation durations and skipping probabilities on the target word (article), the pre-boundary word to the left of the target word, and the post-target word to the right of the target word.

#### 3.2.1. Target Word (Article)

Table 3 shows posterior coefficient estimates and 95% credible intervals (CIs) from Bayesian Linear Mixed Models (BLMMs) fitted to skipping probability, FFD, GD, and TVT on the target word (the article). For both singular and plural articles, skipping percentages differed by less than 3% between the identical and dissimilar preview conditions, and the effect of preview type on the probability of skipping the article was not credible as the 95% credible interval included 0 (b = 0.03, 95% CI [–0.11, 0.18]). This indicates that the parafoveal article–noun mismatch did not substantially affect the likelihood of fixating the article. As expected, singular articles were skipped more often than plural ones, reflecting general effects of word length and visual salience.

When the article was fixated, fixation durations were longer following dissimilar previews compared to identical ones (≈15–30 ms increase). This preview effect was not credible in first fixation duration (β = 0.02, 95% CI [−0.01, 0.04]) or gaze duration (β = 0.04, 95% CI [−0.01, 0.09]). In contrast, total viewing times on the article were longer following dissimilar previews (β = 0.06, 95% CI [0.02, 0.10]), suggesting that the parafoveal information did interfere with later processing and, possibly, syntactic integration of the article and the noun.

Across conditions, plural forms showed overall longer fixation durations and lower skipping probabilities than singular forms (Skipping: β = 0.574, 95% CI [0.424, 0.724]; GD: β = −0.072, 95% CI [−0.120, −0.023]; TVT: β = −0.095, 95% CI [−0.139, −0.050]). This is consistent with typical effects of word length and morphological complexity. No reliable effects of target gender or interactions were observed, as all corresponding credible intervals included zero.

#### 3.2.2. Pre-Boundary Word

Bayesian linear mixed-effects models were also fitted for the pre-boundary word (the word preceding the article) to assess potential parafoveal-on-foveal effects of the preview manipulation. Table 4 summarizes posterior estimates and 95% credible intervals for skipping probability, first fixation duration (FFD), gaze duration (GD), and total viewing time (TVT). No credible effects of preview condition on fixation times on the pre-boundary word were observed on any measure, indicating that the manipulation applied to the upcoming article did not influence fixation behavior on the preceding word. Skipping probability and fixation durations were comparable across identical and dissimilar preview conditions.

A credible main effect of target gender on the pre-boundary word was observed for total viewing time (β = −0.106, 95% CI [−0.204, −0.011]), with slightly shorter total viewing times on articles preceding masculine compared to feminine nouns, regardless of the preview condition. It is not clear what causes this small effect, as the noun was still relatively far away while participants were fixating the article. It may be related to the status of masculine as the default grammatical gender in Spanish, but in order for this to be possible participants would need to start processing the noun already while fixating on the pre-boundary word. No other effects or interactions were supported by the data, as all corresponding credible intervals included zero.

These results confirm that the preview manipulation had no measurable influence on the processing of the pre-boundary region, establishing a stable baseline for interpreting effects on the article and the subsequent noun.

#### 3.2.3. Post-Target Word (Noun)

Bayesian linear mixed-effects models were also fitted for the post-target region (the noun) to assess potential spillover effects of the parafoveal preview manipulation. Table 5 summarizes posterior estimates and 95% credible intervals for skipping probability, first fixation duration (FFD), gaze duration (GD), and total viewing time (TVT).

A credible main effect of preview condition was found for total viewing time (β = 0.040, 95% CI [0.013, 0.068]), indicating that nouns were read for longer following dissimilar previews compared to identical ones. This suggests that parafoveal mismatches between article and noun led to increased processing demands on the subsequent word.

Additionally, a credible interaction between number and preview condition was observed for first fixation duration (β = 0.035, 95% CI [0.006, 0.065]), with larger preview effects for plural compared to singular forms. This may reflect the larger visual impact of the display change from “la” to “el” and vice versa compared to the change between “los” and “las”. Alternatively, it may reflect the fact that the shorter singular articles were faster to process parafoveally than the longer plural articles, leading to a larger effect for the singular articles. In any case, this interaction was only credible in FFD, suggesting that this effect was transitory and largely irrelevant in later processing.

A strong and credible main effect of number was also found across measures (Skipping: β = 0.817, 95% CI [0.388, 1.216]; GD: β = −0.039, 95% CI [−0.073, −0.006]; TVT: β = −0.057, 95% CI [−0.091, −0.022]), indicating that plural nouns were fixated less often and for shorter durations overall than singular ones.

No other credible main effects or interactions were observed, as all corresponding credible intervals included zero. Overall, these findings indicate that while parafoveal agreement violations did not broadly affect skipping or early measures, they produced measurable downstream effects on total viewing time, consistent with a delayed integration cost on the noun.

## 4. Experiment 2

Experiment 2 was designed to replicate and extend the findings of Experiment 1 using a different pair of articles in Spanish: *una* (feminine singular indefinite article *a* in English) and *los* (masculine plural definite article *the* in English). These two articles were selected for their specific grammatical features—contrasting in both gender and number—as well as for their identical length, which is crucial for preserving the integrity of the gaze-contingent boundary paradigm. By keeping the length of the article constant across conditions, we ensured that display changes did not create noticeable shifts in word position, which could otherwise alert participants to the manipulation and invalidate the parafoveal preview effect.

### 4.1. Method

#### 4.1.1. Participants

There were 24 participants (19 women and 6 men) with a mean age of 24.64 years. None of the participants had participated in Experiment 1 and all were naïve to the manipulation and objective of the experiment. The inclusion criteria, recruitment procedures, and compensation were identical across both experiments. The data of five additional participants who reported seeing more than ten display changes were removed.

#### 4.1.2. Apparatus

The experimental setup, including the eye-tracking equipment, calibration procedures, and display settings, were identical to those used in Experiment 1.

#### 4.1.3. Materials

We constructed 210 sentences in Spanish, different from Experiment 1 but following similar criteria: half of which contained a feminine singular noun (e.g., *mesa*) and the other half a masculine plural noun (e.g., *vinos*). Each noun was selected from the EsPal database and matched for high lexical frequency and length as it was in Experiment 1. Singular nouns were between 4 and 6 letters long, and plural nouns between 4 and 7 letters, accounting for the additional plural suffix. Each sentence included a mid-sentence article–noun phrase (e.g., *una mesa*, *los vinos*), and a gaze-contingent boundary was placed at the end of the word preceding this phrase.

The preview manipulation consisted of two conditions: In the correct preview condition, the article presented parafoveally matched the upcoming noun in gender and number (e.g., *una mesa*, *los vinos*). In the dissimilar preview condition, the article presented was incongruent with the noun, such that *una* was replaced with *los* and vice versa (e.g., los* mesa, una* vinos). Once a participant’s gaze crossed the boundary, the article was replaced with the grammatically correct form. Table 6 shows examples of each preview condition for the sentences with feminine/singular and the sentences with masculine/plural nouns. Appendix A.2 contains all experimental sentences used in Experiment 2.

As in Experiment 1, an additional 10 practice sentences were included at the beginning of the session, resulting in each participant reading a total of 220 sentences.

#### 4.1.4. Design

The experiment employed a within-subjects and within-items design with two two-level factors: Preview condition (identical vs. dissimilar) and target article type (*una* vs. *los*), manipulated between items but within subjects.

Each of the 210 sentences was divided into three blocks of 70. Within each block, 35 sentences contained a feminine singular noun and 35 a masculine plural noun. The article preview was manipulated parafoveally as described above.

Block order was counterbalanced across participants and preview condition (identical vs. dissimilar) was also counterbalanced so that each participant saw every item in only one condition. Across the sample, all items appeared equally often in each condition. 

#### 4.1.5. Procedure

The trial structure, task instructions, and timing of sentence presentation followed the same procedure as in Experiment 1. Participants were instructed to read for comprehension and respond to occasional yes/no questions about sentence content. Each participant completed three blocks of trials with short breaks in between to prevent fatigue.

#### 4.1.6. Data Analysis

Data processing, exclusion criteria, and statistical analysis methods followed the same procedures as outlined in Experiment 1.

In all cases, the model included the fixed effects of preview (identical vs. dissimilar), target number/gender (feminine singular vs. masculine plural) as well as their interaction and a maximal random effects structure as in Experiment 1.

The same preprocessing and exclusion criteria were applied as in Experiment 1. After filtering, 4.48% of the fixations were excluded based on these criteria. Specifically, 2.84% were long fixations (>800 ms), 1.1% were removed due to proximity to blinks, and 0.56% were short fixations (<80 ms).

### 4.2. Results and Discussion

#### 4.2.1. Target Word (Article)

Skipping rates for the article were generally similar across preview conditions. Table 7 presents mean fixation durations and skipping probabilities for the pre-boundary word, target (article), and post-target (noun) regions as a function of preview condition and grammatical number–gender combination. Table 8 summarizes posterior estimates and 95% credible intervals for skipping probability, first fixation duration (FFD), gaze duration (GD) and total viewing time (TVT) from the BLMMs fitted. No credible effects of preview condition were found for skipping probability or early fixation measures (FFD, GD), as all credible intervals included zero. However, a credible effect of preview condition was observed for total viewing time (β = 0.077, 95% CI [0.015, 0.137]), indicating that articles were read for longer when the preview mismatched the grammatical features of the upcoming noun.

Overall, these results show that parafoveal mismatches did not influence saccade targeting or early lexical processing of the article but led to longer overall reading times, suggesting increased integration effort during later processing stages.

#### 4.2.2. Pre-Boundary Word (Word Preceding the Article)

Table 9 summarizes posterior estimates and 95% credible intervals for pre-boundary word skipping probability, first fixation duration (FFD), gaze duration (GD) and total viewing time (TVT) from the BLMMs fitted. No credible effects of preview condition were found for skipping probability or early fixation measures (FFD, GD), as all credible intervals included zero. However, as on the article a credible positive effect of preview condition was observed for total viewing time (β = 0.036, 95% CI [0.006, 0.066]), showing that the later processing difficulty induced by the preview extended not just to the article itself, but also to re-reading the pre-boundary word.

#### 4.2.3. Post-Target Word (Noun)

Table 10 summarizes posterior estimates and 95% credible intervals (CIs) for post-target word skipping probability, first fixation duration (FFD), gaze duration (GD), and total viewing time (TVT) from the Bayesian linear mixed models. No credible effects of preview condition were found for skipping probability or gaze duration, as the 95% CIs included zero. A small but credible effect of preview condition was observed for First Fixation Duration on the post-target word (β = 0.021, 95% CI [0.001, 0.040]). Also, as on the article a credible positive effect of preview condition was observed for total viewing time (β = 0.051, 95% CI [0.013, 0.088]), showing that the effect of the dissimilar preview condition extended to the noun region, increasing the time readers spent processing it.

A strong main effect of target number/gender was found for skipping probability (β = 1.036, 95% CI [0.497, 1.652]), with plural forms being less likely to be skipped than singular ones. Additionally, credible negative effects of number/gender were observed for gaze duration (β = −0.063, 95% CI [−0.106, −0.021]) and total viewing time (β = −0.058, 95% CI [−0.103, −0.012]), indicating overall longer fixation durations for plural compared to singular targets. No credible interactions were observed between preview condition and target number/gender, as all corresponding credible intervals included zero.

Across all regions, the results of Experiment 2 showed a consistent pattern, which was very similar to Experiment 1: parafoveal grammatical mismatches between the article and the noun did not influence skipping probability or early fixation measures on the article but produced small and reliable effects in later measures and on the noun.

In the pre-boundary region, only a minor effect was found on total viewing time, suggesting minimal parafoveal-on-foveal influence. In the target region (the article), total viewing time was longer following dissimilar previews, indicating that mismatching grammatical information increased late-stage processing demands. Finally, in the post-target region (the noun), the same pattern was observed, with longer first fixation durations and total viewing times consistent with a spillover effect of the preview manipulation.

Overall, this suggests that readers take neither gender nor number agreement into account when making the decision of whether to skip an article or not.

## 5. General Discussion

This study investigated whether grammatical agreement between parafoveal articles and nouns affects eye movements during Spanish sentence reading. Across two experiments, presenting a preview of an article whose gender and, in Experiment 2, number did not agree with the subsequent noun did not appear to have a meaningful influence on early reading behaviors such as skipping or first fixation duration. Instead, effects primarily emerged later—mainly in total viewing time on the article and the following noun. This pattern suggests that, while the parafoveal preview information is not completely ignored, readers do not seem to use it to guide eye movements. Rather, the information from the preview seems to affect later, potentially post-lexical, processes such as error checking and syntactic integration. This timing is largely consistent with earlier reports of delayed syntactic effects in agreement and dependency studies [34,35].

*Parafoveal information about articles causes delayed disruption.* The lack of early disruption may be surprising at first glance, given that our manipulation involved presenting an ungrammatical sequence of words on the screen (albeit only parafoveally). However, it fits in with Staub et al.’s [12] recent work demonstrating that readers often fail to notice ungrammatical repetitions or omissions of short function words, and that when such errors are not consciously detected, they do not disrupt eye movements. Rather than reflecting an absence of processing, this pattern suggests that readers can perceptually “correct” deviations in the input at an early stage of processing, maintaining a coherent sentence representation. From this perspective, the absence of early effects of article–noun mismatches in our data may be because the perceptual anomaly of seeing wrong article may not be enough to influence eye-movement planning. It may be sufficient for readers to confirm that the upcoming word is compatible with being an article in order to skip it. Perceptual inference could therefore act as a compensatory mechanism that preserves fluent reading at the cost of temporarily masking syntactic inconsistencies. This interpretation aligns with the noisy-channel model of sentence comprehension [36] which posits that the comprehension system prioritizes plausible and internally consistent representations even under uncertain or degraded input. The fact that we did observe evidence for later disruption shows that the parafoveal information about the article is not completely ignored. Instead, it seems that the parafoveal information about the article is not fully evaluated and incorporated into the perceptual inference process at least until the noun is processed. Given that, even on the noun, we do not observe these effects until late, the disruption likely arises during the final integration stages.

*Informational economy.* Our results paint a slightly different picture of word identification during reading compared to existing serial and parallel models: Just as Angele and Rayner [9] observed, the parafoveal information about articles does not have to be processed exhaustively to trigger skipping them. Rather, it may be that readers can “hold off” on completely identifying very short high-frequency words and defer their integration until the end of the clause or sentence is reached. We observed the same disruption effects on the post-target word as Angele and Rayner [9] and Abbott et al. [11], but, in our case, the effects only emerged in TVT. This is likely because the anomaly in the present study was much more subtle (an agreement violation) than in those studies (a change in part of speech, with the article preview appearing where a three-letter verb should be). This difference gives us an idea of how error detection and error correction operate during reading: processing can continue uninterrupted even if there is some uncertainty about which article was seen, but when new information suggests that a verb was misread as an article, this requires immediate error correction. The fact that skipping probability is not affected by grammatical incongruency suggests that the oculomotor system may operate under a principle of informational economy. As noted by Viera et al. [37], articles carry minimal semantic load and serve a primarily grammatical function. Consequently, when an inconsistency is detected parafoveally, it may be inefficient for the reader to devote an additional fixation to such a short and highly predictable function word.

Rather than interrupting processing at the article, the reading system appears to prioritize advancing to the noun, which constitutes the informational core of the phrase and carries the information necessary to resolve any syntactic ambiguity. This behavior is consistent with the idea that readers may “postpone” the full integration of high-frequency words, processing them more shallowly in order to maintain an efficient reading rate. From this perspective, skipping the article—even when it is grammatically incorrect—can be interpreted as a rational strategy that favors access to lexically informative content over the immediate correction of minor functional errors.

*Serial* vs. *parallel processing.* Within the broader debate on serial versus parallel models of eye-movement control, these findings suggest that parafoveal processing may operate at multiple levels simultaneously: while lexical and visual information guide early saccade targeting, perceptual inference mechanisms may intervene to maintain fluency when syntactic inconsistencies arise. Our results can be contrasted with recent findings from Brazilian Portuguese, where articles appear to be skipped less often when they carry specific morphological or informational weight [37]. In addition, recent evidence presented by Viera et al. [38] suggests that the parafoveal information about articles seems to be mostly ignored during reading in Brazilian Portuguese, even during the subsequent processing of the noun. In Spanish, by contrast, it seems that some parafoveally presented information about the article does linger in working memory and affects late processing of the noun. This may simply be because Spanish articles are longer and take up more of the visual field than Portuguese articles. It may be easier to resolve conflicts due to lingering parafoveal information in Portuguese just because the shorter Portuguese articles are less visually salient than the Spanish ones.

Considering how our results fit in with existing models of reading, the lack of effects of ungrammatical previews is consistent with models positing largely serial lexical processing, such as E-Z Reader [21], as the lack of agreement between the article and the noun cannot be detected if the noun has not been processed parafoveally. However, this result would also be compatible with parallel models that assume that syntactic information is not processed immediately but rather integrated at the end of a clause. In fact, E-Z Reader 10 [18] allows for integration failures that trigger regressions to previous parts of the sentence but does not contain a theoretical or computational account of what causes these integration failures. On the other hand, parallel models like SWIFT [39] and OB1-Reader [40] make few claims about syntactic integration.

*Possible theoretical accounts.* In contrast, the rational reading model [23,24] is a Bayesian account of the reading process that assumes that regressions can be triggered by uncertainty about the identity of a word. As readers reach and process the noun, identifying its grammatical gender may lead to increased uncertainty about the identity of the article, leading to regressions. However, it is unclear why the effects of this uncertainty would not appear immediately once the noun is fixated rather than late as we observed.

The recent SEAM model (Sentence-Processing and Eye-Movement Activation-Coupled Model, [25]) combines SWIFT and the Lewis and Vasishth sentence processing model (LV05, [26]). In LV05, sentence reading times are influenced by the need to perform memory retrieval operations. These operations are triggered by syntactic dependencies. For example, processing a verb will trigger a search in working memory for a word that can serve as the subject. In the present study, both processing the noun and processing subsequent verbs may lead to a retrieval operation that could be disrupted by the incorrect article being stored in working memory. For example, in the sentence “El genio cumplirá el deseo que pidas.” (“The genie will fulfill the wish that you make”), processing the word *deseo* may lead to a search for the corresponding article, which, in the dissimilar preview condition, then would fail since *la* has different semantic features compared to *el*. Processing the verb *pidas* (make) at the end of the sentence may trigger a retrieval operation for the object *el deseo* (the wish), which could also be disrupted by *la* not matching the syntactic features of *deseo*. The disrupted retrieval process would then trigger regressions to the source of the difficulty, accounting for the increased total viewing times we observed. It is important to note that SEAM currently only implements retrieval based on verbs, which means that it may not be able to account for all of our observations.

Deferred processing of the article would be a sensible strategy to avoid confusion in German or Dutch, where the same articles are used for nouns with different grammatical gender, number, and case, causing potential ambiguity. However, Spanish articles are completely transparent regarding gender and number. The absence of early effects even in a completely transparent system suggests that the deferred parafoveal processing of articles we observed is universal when reading short high-frequency words in any language rather than modulated by particular syntactic features of a language. In effect, processing an article that occurs tens of thousands of times per million [6] has likely reached a level of automatization where the fact that the upcoming word is an article is more important than which article it is. This also fits in with our observation that the stronger differences in the visual shape of the dissimilar preview for singular articles compared to plural articles *(el/la* vs. *los/las*) did not seem to lead to earlier detection of the anomaly or stronger disruption. Future work combining eye-tracking and electrophysiological measures could offer a better timeline of processing of syntactic information during reading.

*Conclusions.* Overall, presenting readers with syntactically incorrect articles in the parafovea clearly has consequences, but these consequences do not become apparent in the eye movement record until the late stages of sentence processing. As none of the “classic” serial and processing models of eye-movement control provide strong theoretical or computational accounts of syntactic processing during reading, they cannot explain these effects. However, the SEAM model [25], which combines parallel word identification with memory retrieval in post-lexical processing, may offer some clues as to how these results can be explained, as can the Bayesian rational reading model [23,24]. Overall, it seems that readers can defer some processing during first-pass reading, especially of short high-frequency words, and then use perceptual inference to arrive at a coherent representation of a sentence. It is at this point where conflicting information that had been presented in the parafovea can affect the processing of the sentence. Such a strategy could enable readers to maintain a high reading rate while at the same time being robust to perceptual errors—such as mistaking *el* for *la* or *los* for *las.*

## Figures and Tables

**Table 1:** Example sentences for each condition.

Gender	Number	Identical Preview	Dissimilar Preview
Feminine	Singular	“He olvidado limpiar *la* sala del fondo.”	“He olvidado limpiar el* sala del fondo.”
	Plural	“He olvidado limpiar *las* salas del fondo.	“He olvidado limpiar los* salas del fondo.
Masculine	Singular	“El genio cumplirá *el* deseo que pidas.”	“El genio cumplirá la* deseo que pidas.”
	Plural	“El genio cumplirá *los* deseos que pidas.”	“El genio cumplirá las* deseos que pidas.”

English translation of the sentences: “I forgot to clean the room(s) at the back” (feminine); “The genie will fulfill the wish(es) you make” (masculine). Ungrammatical previews are marked by asterisks which were not shown on the screen.

**Table 2:** Condition means for fixation durations and skipping probabilities (Experiment 1).

			Pre-Boundary Word				Target (Article)				Post-Target (Noun)			
Number	Noun Gender	Preview	FFD (ms)	GD (ms)	TVT (ms)	Skipping (%)	FFD (ms)	GD (ms)	TVT (ms)	Skipping (%)	FFD (ms)	GD (ms)	TVT (ms)	Skipping (%)
singular	feminine	identical	194	409	487	31.6	181	284	337	64.0	200	417	505	11.7
singular	feminine	dissimilar	193	421	494	32.2	188	306	367	62.7	205	429	544	9.0
singular	masculine	identical	192	412	513	28.1	176	285	343	65.1	198	414	535	10.2
singular	masculine	dissimilar	199	448	562	26.0	188	303	349	68.1	205	432	567	10.3
plural	feminine	identical	193	405	477	33.8	182	303	367	50.8	194	432	563	5.6
plural	feminine	dissimilar	193	410	495	33.8	187	333	396	53.8	192	432	577	4.4
plural	masculine	identical	196	451	534	25.2	184	298	364	55.7	198	440	568	4.6
plural	masculine	dissimilar	195	430	519	25.4	184	317	397	53.8	193	441	566	5.4

**Table 3:** Experiment 1: Bayesian linear mixed-effects model estimates and 95% credible intervals (CIs) for the target word (article). Coefficients for which the CIs did not include 0 are shown in bold.

	Skipping	FFD	GD	TVT
Intercept	**0.466**	**5.163**	**5.599**	**5.710**
	[**0.221, 0.704**]	[**5.098, 5.232**]	[**5.522, 5.679**]	[**5.632, 5.786**]
Preview condition	0.034	0.018	0.044	**0.059**
	[−0.112, 0.177]	[−0.006, 0.042]	[−0.007, 0.091]	[**0.017, 0.099**]
Number	**0.574**	−0.007	**−0.072**	**−0.095**
	[**0.424, 0.724**]	[−0.037, 0.024]	[**−0.120, −0.023**]	[**−0.139, −0.050**]
Noun gender	−0.144	0.011	0.026	0.031
	[−0.417, 0.137]	[−0.017, 0.039]	[−0.026, 0.081]	[−0.026, 0.089]
Number × Preview condition	0.017	0.020	−0.007	0.001
	[−0.283, 0.322]	[−0.029, 0.068]	[−0.086, 0.070]	[−0.079, 0.080]
Number × Noun gender	−0.043	0.017	−0.003	0.011
	[−0.307, 0.223]	[−0.028, 0.062]	[−0.081, 0.075]	[−0.071, 0.089]
Preview × Number × Gender	−0.447	−0.031	−0.015	−0.004
	[−1.028, 0.133]	[−0.133, 0.076]	[−0.171, 0.143]	[−0.167, 0.164]

**Table 4:** Experiment 1: Bayesian linear mixed-effects model estimates and 95% CIs for the pre-boundary word. Coefficients for which the CIs did not include 0 are shown in bold.

	Skipping	FFD	GD	TVT
Intercept	**−1.878**	**5.213**	**5.843**	**5.983**
	**[−2.342, −1.430]**	**[5.149, 5.280]**	**[5.756, 5.929]**	**[5.889, 6.076]**
Preview condition	−0.058	0.007	0.003	0.030
	[−0.307, 0.170]	[−0.011, 0.025]	[−0.036, 0.043]	[−0.003, 0.064]
Number	0.029	0.002	−0.008	0.013
	[−0.179, 0.249]	[−0.015, 0.020]	[−0.043, 0.028]	[−0.020, 0.045]
Noun gender	0.583	−0.012	−0.073	**−0.106**
	[−0.186, 1.353]	[−0.037, 0.014]	[−0.149, 0.004]	**[−0.204, −0.011]**
Number × Preview condition	−0.134	0.004	0.052	0.043
	[−0.573, 0.266]	[−0.034, 0.042]	[−0.020, 0.125]	[−0.020, 0.105]
Number × Noun gender	−0.346	0.003	0.036	0.017
	[−0.726, 0.030]	[−0.033, 0.039]	[−0.029, 0.099]	[−0.045, 0.078]
Preview × Number × Gender	0.323	−0.061	−0.106	−0.115
	[−0.432, 1.101]	[−0.131, 0.010]	[−0.251, 0.035]	[−0.241, 0.013]

**Table 5:** Experiment 1: Bayesian linear mixed-effects model estimates and 95% CIs for the post-target word (noun). Coefficients for which the CIs did not include 0 are shown in bold.

	Skipping	FFD	GD	TVT
Intercept	**−3.372**	**5.239**	**5.952**	**6.166**
	**[−4.023, −2.760]**	**[5.172, 5.307]**	**[5.875, 6.027]**	**[6.069, 6.265]**
Preview condition	−0.107	0.003	0.018	0.040
	[−0.417, 0.209]	[−0.014, 0.020]	[−0.009, 0.044]	[0.013, 0.068]
Number	**0.817**	0.025	**−0.039**	**−0.057**
	**[0.388, 1.216]**	[−0.000, 0.049]	**[−0.073, −0.006]**	**[−0.091, −0.022]**
Noun gender	0.013	0.000	−0.002	−0.015
	[−0.322, 0.367]	[−0.021, 0.021]	[−0.038, 0.035]	[−0.062, 0.032]
Number × Preview condition	−0.266	**0.035**	0.021	0.044
	[−0.969, 0.429]	**[0.006, 0.065]**	[−0.030, 0.072]	[−0.013, 0.102]
Number × Noun gender	0.072	0.018	0.030	−0.033
	[−0.507, 0.691]	[−0.015, 0.050]	[−0.024, 0.084]	[−0.094, 0.026]
Preview × Number × Gender	−0.125	−0.030	−0.021	−0.043
	[−1.449, 1.042]	[−0.109, 0.050]	[−0.138, 0.099]	[−0.201, 0.111]

**Table 6:** Example sentences for each condition in Experiment 2.

Gender	Number	Identical Preview	Dissimilar Preview
Feminine	Singular	“Hoy me compré una camisa muy bonita.”	“Hoy me compré los* camisa muy bonita.”
Masculine	Plural	“Este mercado tiene los precios más baratos de la ciudad.”	“Este mercado tiene una* precios más baratos de la ciudad.”

English translation of the sentences: “Today I bought a very beautiful shirt” (feminine); “This market has the cheapest prices of the city” (masculine). Ungrammatical previews are marked by asterisks which were not shown on the screen.

**Table 7:** Experiment 2: Means for fixation durations and skipping probabilities by preview condition and target word number/gender.

	Experiment 2—Summary Means by Position and Measure													
	Factors: Number—Gender × Preview													
			Pre-Boundary Word				Target (Article)				Posttarget (Noun)			
Article	Number–Gender	Preview	FFD (ms)	GD (ms)	TVT (ms)	Skipping (%)	FFD (ms)	GD (ms)	TVT (ms)	Skipping (%)	FFD (ms)	GD (ms)	TVT (ms)	Skipping (%)
Una	Singular/feminine	identical	195	467	600	4.5	178	275	323	60	201.2	435	539	6.5
Una	Singular/feminine	dissimilar	199	478	612	5.3	186	289	350	56	206.7	445	574	7.4
Los	Plural/masculine	identical	196	467	595	8.8	178	284	331	55	198.8	464	592	3.4
Los	Plural/masculine	dissimilar	195	474	592	7.1	180	286	353	59	203.7	479	608	3.1

**Table 8:** Experiment 2: Bayesian linear mixed-effects model estimates and 95% CIs for the article (target word). Coefficients for which the CIs did not include 0 are shown in bold.

	Skipping	FFD	GD	TVT
Intercept	0.370	**5.151**	**5.548**	**5.649**
	[−0.008, 0.750]	[**5.094, 5.204**]	[**5.478, 5.620**]	[**5.559, 5.738**]
Preview condition	−0.022	0.023	0.027	**0.077**
	[−0.153, 0.109]	[−0.002, 0.048]	[−0.014, 0.068]	[**0.015, 0.137**]
Noun Number/Gender	0.040	0.008	−0.014	−0.009
	[−0.154, 0.231]	[−0.017, 0.033]	[−0.058, 0.029]	[−0.057, 0.039]
Number/Gender × Preview condition	**−0.341**	0.017	0.012	−0.006
	[**−0.611, −0.073**]	[−0.034, 0.067]	[−0.072, 0.093]	[−0.094, 0.079]

**Table 9:** Experiment 2: Bayesian linear mixed-effects model estimates and 95% CIs for the pre-boundary word. Coefficients for which the CIs did not include 0 are shown in bold.

	Skipping	FFD	GD	TVT
Intercept	**−4.782**	**5.238**	**6.015**	**6.219**
	[**−5.542, −4.092**]	[**5.184, 5.294**]	[**5.931, 6.099**]	[**6.104, 6.341**]
Preview condition	−0.134	0.005	0.017	**0.036**
	[−0.753, 0.425]	[−0.011, 0.021]	[−0.015, 0.049]	[**0.006, 0.066**]
Noun Number/Gender	−0.374	0.005	0.013	0.047
	[−1.315, 0.552]	[−0.016, 0.025]	[−0.039, 0.066]	[−0.022, 0.116]
Number/Gender × Preview condition	−0.152	0.021	−0.007	0.002
	[−1.625, 1.029]	[−0.012, 0.055]	[−0.084, 0.069]	[−0.061, 0.067]

**Table 10:** Experiment 2: Bayesian linear mixed-effects model estimates and 95% CIs for the post-target region. Coefficients for which the CIs did not include 0 are shown in bold.

	Skipping	FFD	GD	TVT
Intercept	**−3.708**	**5.275**	**6.004**	**6.216**
	[**−4.254, −3.211**]	[**5.224, 5.326**]	[**5.921, 6.087**]	[**6.101, 6.326**]
Preview condition	0.034	**0.021**	0.019	**0.051**
	[−0.402, 0.422]	[**0.001, 0.040**]	[−0.014, 0.053]	[**0.013, 0.088**]
Noun Number/Gender	**1.036**	0.011	**−0.063**	**−0.058**
	[**0.497, 1.652**]	[−0.006, 0.028]	[**−0.106, −0.021**]	[**−0.103, −0.012**]
Number/Gender × Preview condition	0.331	−0.001	−0.014	0.009
	[−0.467, 1.178]	[−0.032, 0.030]	[−0.071, 0.042]	[−0.047, 0.066]

## Data Availability

The original data presented in the study are openly available in OSF at https://osf.io/2zf4p/.

## Appendix A

### Appendix A.1. Sentences Experiment 1

Los cuadernos estaban llenos de dibujos hechos por la niña pequeña.
Se educado con la visita esta tarde.
Si veo películas de miedo por la noche tengo pesadillas.
Si le estás buscando, debe estar por la villa del final de la calle.
No me gusta vagabundear por la calle de noche.
No puedo olvidar la carta que me escribiste.
Sigue negando el acto delictivo.
El árbol fue alcanzado por el rayo y empezó a arder.
No confundas el precio con la calidad.
Siempre está diciendo que las cosas estaban mejor en el siglo pasado.
Tuvo problemas con el gato y ya no quiere ninguno.
La niña no ha superado el miedo nocturno.
El arquitecto criticó las formas de trabajar del albañil.
En este mapa no puedo ver las zonas que dices.
Para aprobar solo tienes que dominar las sumas y nada más.
Eso es erróneo según las teorías más actuales.
Estamos todos juntos en las luchas por un mundo más justo.
Este juez imparte siempre las penas más duras.
Hablamos con los autores durante la firma de libros.
En la excursión al museo analizamos los estilos artísticos de las pinturas.
La noticia recorrió los países causando gran conmoción.
Borré el archivo con los títulos de los libros.
Para comenzar la mañana preparé los cafés bien fuertes.
Este sábado iremos de excursión con los padres de mi vecino.
Ve aminorando la marcha poco a poco antes de detenerte.
No puedo mirar la cara que ponen.
Hay que tener en cuenta la figura materna en la infancia.
Nos hemos organizado para que la comida sea a su hora.
Dedicó toda su vida a escribir la novela de fantasía.
Este libro contiene la poesía más bonita que he leído nunca.
Mañana visitaremos la sede para discutir el plan de expansión.
Asistieron al evento con la esposa y los hijos.
Era fácil admirar la fuente del centro de la plaza.
Tenemos que trabajar con la unidad de salvamento.
Después de entrenar estamos listas para la carrera solidaria.
Era una cena especial, así que arreglamos la mesa elegantemente.
En clase de lengua damos el medio de comunicación y el lenguaje.
Es un especialista, ha hecho el marco más espectacular.
Cada vez se fomenta menos la excelencia en el ámbito académico.
El abogado dejó el objeto sobre la mesa.
Mi abuelo miraba el álbum de fotos con nostalgia.
En mi sueño estaba volando por el aire y veía las ciudades debajo de mí.
Mi tío preparó el texto que debíamos leer.
Lo he metido todo en el fondo de inversión.
Para tomar una decisión consúltalo con el jefe antes de nada.
Desarrollamos nuevas habilidades para ascender en el cargo de la empresa.
Estamos todavía eligiendo el color para pintar las paredes.
Es importante establecer un acuerdo con las partes implicadas.
No te entrometas en las vidas de los demás.
He visto a los bomberos por las calles del pueblo.
No podemos ignorar las muertes de tanta gente.
Todo parece indicar que las obras acabarán en el momento pactado.
El camarero se enfadó con las mujeres sin motivo.
Este fin de semana planeo terminar las series que estaba viendo.
Me casé para quedarme con las tierras de mi marido.
El lechero deja las botellas en la puerta por las mañanas temprano.
El millonario dio a elegir entre las cajas sorpresa o un millón de euros.
Es muy bonito sentirse uno más entre las gentes del lugar.
Espero que las cosas salgan como planeamos.
No perdamos de vista los tiempos del marcador.
Si vamos con los señores no llegaremos a tiempo.
A través de los libros exploramos los mundos desconocidos.
No me quiere decir los lugares que vamos a visitar.
No me sé los nombres de nadie aquí.
Quién iba a decir que los hombres llegarían tan lejos.
Esta tarde juntamos los grupos de amigos.
Debemos averiguar cómo anular los poderes del mago.
Creo que si miras los puntos fijamente empezarás a ver que se mueven.
Para hacer las cortinas necesito saber los tipos de tela que quieres.
En clase estamos estudiado sobre los estados de la materia.
Disfruté mucho al dar la vuelta en la noria.
No puedo vencerte con la fuerza que tengo.
Espero que no cambien la fecha del examen a estas alturas.
Todas las semanas riego la planta que tengo en el salón.
Está seguro de que la imagen que presenta ganará el concurso.
El satélite capta la luna perfectamente.
No quiero quedarme en la ciudad durante el verano caluroso.
Jugando a las cartas pasamos el rato en el pueblo.
Entregamos el proyecto en el plazo previsto.
Luego quedamos por el puesto de perritos.
Me asusté muchísimo esta noche cuando escuché el golpe contra la pared.
Se me olvidó atar el cabo al barco.
En clase de historia estamos haciendo un trabajo sobre las guerras actuales.
Se nota que es calmado por las maneras de hablar que tiene.
Hay mucha producción de aceite en las regiones del sur.
He olvidado limpiar las salas del fondo.
En el centro de la ciudad puedes encontrar las plazas más antiguas.
Tengo clases de inglés por las tardes a última hora.
El documental va sobre los juicios del año pasado.
No puedo más, voy a tomarme la medicación para los dolores de espalda.
Están haciendo reformas por los barrios de la periferia.
He acabado de limpiar los suelos de esta planta.
No juzgues tanto los motivos de la gente.
Espero que no le retiren los apoyos o se quedará solo.
Ya no se hace el pan como en la década pasada.
Debemos tener en cuenta la salida de emergencia.
El dentista debe estar cansado de ver todo el día la boca de la gente.
Mi tío está preocupado por la piedra del riñón.
No encuentro la página que comentó el profesor.
Podremos hablar con la mente que ha desarrollado el proyecto.
Ha cerrado la venta de forma muy eficiente.
Como siempre la labor recae sobre mí.
No puedes tú solo con la carga de la mudanza.
No podemos sentarnos con la reina de la belleza.
La calidad no siempre reside en la marca de la ropa.
Hay que abordar el asunto con tacto.
Con la autorización obtuvimos el acceso a la base de datos.
Después de esta entrevista conseguiremos el empleo seguro.
Siempre tiene una excusa para fastidiar el plan a todos.
El genio cumplirá el deseo que pidas.
Para aprovechar el cuarto organiza mejor el espacio.
Para el estreno visitamos antes el cine y sus salas.
Me estoy estresando mucho con el viaje de este verano.
La guía muestra el sitio más destacado de la ciudad.
Me aterra que suban el grado de dificultad.
Mi padre no quiere asumir el riesgo de la operación.
El ladrón esquivó el ataque del policía.
En el congreso podremos hablar con las cabezas más brillantes de este ámbito.
Hoy es un día muy importante para las madres de todos.
Estoy seguro de que tengo las razones necesarias para tomar esta decisión
Mi abuela suele decir que a veces las verdades duelen mucho.
Me pregunto si las horas han cambiado.
Sin un buen equipo las ideas no llegarían a ninguna parte.
Mi abuelo habla mucho sobre las épocas que pasó en el campo.
El pensamiento va antes que las acciones casi siempre.
Hay que esperar unos minutos antes de quitar las bandas de cera.
Al final me quede con las dudas por no preguntar.
Algunas personas tienen problemas con las edades de los demás.
Debes confiar en los equipos de programadores.
No podemos ignorar los hechos de la semana pasada.
No puede ser que los hijos tengan que estar a tantas cosas.
En medicina estudias sobre los cuerpos humanos.
No puedo con los cambios de hora.
Esperaré hasta que des los pasos necesarios.
El artista seleccionó los papeles de mejor calidad.
Voy a dejar los debates para luego.
Mi madre demostró los valores necesarios.
Hazme caso, sigue los caminos que te dicte el estómago.
Qué bonito es cuando los amores se corresponden.
Para encontrar una solución analizaremos los orígenes del problema.
El profesor dejó la cuenta sin corregir.
Me han asignado la misión más fácil.
Queremos explorar la costa del sur este verano.
En verano haremos la ruta de montaña que recorrimos año pasado.
Como ya he terminado la tarea voy a salir a dar un paseo.
Para terminar el trámite solo necesitamos la firma de los hijos.
El abuelo fue con el niño de paseo.
Requerirás de estrategia y habilidad para ganar el juego de mañana.
Aún podemos escuchar el disco en el reproductor de vinilos.
No dejes que el perro se coma lo que hay en la mesa.
Mi abuelo construyó el muro para que no se escapen las vacas.
Para mi trabajo de arte quiero pintar el rostro de mis abuelos.
No sé si voy a poder superar las pruebas de acceso.
Estamos esperando pacientemente las ayudas pero parece que no llegan.
Con este frío ya se ve nieve en las cumbres de las montañas.
En este texto es muy fácil detectar las faltas de ortografía.
No hay suficiente espacio en las cárceles del condado.
Con esta guerra las ciudades se han perdido.
Los niños jugaban felices en los campos verdes y soleados.
Las flores combinarán perfectamente con los centros de mesa.
Para saber cuánto es pon los pesos sobre la balanza.
Me veo fatal con los pelos así de encrespados.
Nadie se esperaba que los casos de corrupción fueran archivados.
Compré un boleto para la lotería, pero fallé los números finales y no gané nada.
En la conferencia sólo hablaban de encontrar la clave del éxito.
Mañana podremos ver la nave despegar hacia el espacio.
Cada vez está más de moda llevar abrigos con la piel de animales.
Todos los perros son bonitos sin importar la raza que sean.
Antes de salir de casa hice la cama de todos.
La semana que viene tengo la cita con el médico.
Tuvimos que formar la cola de forma ordenada para entrar.
El banco anunció la tasa de interés para préstamos.
Fuimos al teatro con la pareja de recién casados.
No creo que esta economía pueda soportar la caída de la bolsa mucho tiempo.
Está pasando por la fase más intensa del duelo.
Mi abuela no quiere escuchar nada sobre el dios griego del panteón.
El alcalde se perdió al contar el voto electoral.
La cantante domina perfectamente el tono de voz.
Que no se me olvide hacer el pago de fin de mes.
Está esperando el tren que lleva a la ciudad.
Esta tarde vamos a mirar el piso de la agencia.
Quiero ir a bañarme en el lago esta tarde.
Por fin ha asumido el daño que ha hecho.
Iremos a escalar el pico estas vacaciones.
Mi madre no puede con el olor del queso.
Me encanta ver el teatro antes ir a la representación.
Quiero preguntar por el tamaño de las tartas que tenéis.
Recorrí el pasillo y abrí las puertas rápidamente.
Tengo que tener en cuenta las líneas discontinuas para adelantar.
Habrá un gran evento para celebrar las uniones políticas.
Mi prima estará en casa las semanas antes del bautizo.
Está convencido de que las hijas le están mintiendo.
No voy a poder con las clases de hoy, estoy agotada.
La receta variará mucho según las medidas de los ingredientes.
Las tortugas marinas están en las islas para anidar.
La solidaridad impulsa las causas humanitarias.
Siempre recordarás mejor las lenguas que más practiques.
Desde siempre las prensas sirven para aplastar cosas.
Desde este edificio las vistas son impresionantes.
Están explorando los sectores industriales en busca de oportunidades.
Miramos hacia los lados buscando al oso.
Con este examen espero conseguir los éxitos que merezco.
Me gusta poder ajustar los niveles de dificultad en los videojuegos.
Durante las vacaciones disfrutamos los libros por fin.
Los adultos subestiman los efectos del estrés.
Para una sociedad fuerte es esencial cumplir con los deberes cívicos.
Debemos abordar los temas principales, antes de nada.
Guardé en la nevera los restos de la comida.
En momentos difíciles valoro los consejos de mis amigos.
Hace unos años que los pueblos abandonaron esta práctica.

### Appendix A.2. Sentences Experiment 2

Mi sobrino está intentando renovar una casa a las afueras de la ciudad.
Mi novio me compró una cadena de oro.
Hoy he atrapado una araña muy pequeña.
El policía descubrió una bomba en el edificio.
Mi clave personal contiene una serie de varios números y letras.
Este sábado voy a escalar una colina muy empinada.
El gobierno quemó los libros relacionados con la libertad.
El albañil construyó los pisos de esta calle.
Cuando lleguemos tienes que mirar los trenes de alta velocidad.
Necesito que me envíes los enlaces para descargar los artículos.
Hoy me he limpiado los oídos mediante chorros de agua.
El dibujo presenta una forma poco definida.
Le he producido una pena pasajera a mi madre.
En el trabajo he visto una dama muy inteligente.
Afortunadamente mi padre tiene una salud que muchos desearían.
Mi jefa me pidió que recogiera una cosa de la oficina.
Ayer terminé de leer una novela muy famosa.
La empresa de mi abuelo distribuye los peces de la tienda.
Me pidieron diseñar los lados más difíciles de la escultura.
Nos enseñaron que los humanos somos seres sociales.
Hoy explicaré en qué consisten los premios del torneo.
Mañana me van a operar los brazos en el hospital de la ciudad.
Tengo que cuidar los cerdos de mi vecino.
Esta mañana he regado una planta de mi vecina.
El otro día compré una cinta de embalar.
En el futuro quisiera una esposa que me entienda.
Mi suegro puede distinguir una obra auténtica de una falsa.
Este terreno tiene una tierra muy fértil y maleable.
Mi amigo esculpió una cabeza muy detallada.
La madre superiora será una santa muy venerada.
Te has reído como una amiga de mi colegio.
Los libros siempre fueron una fuente de conocimiento.
Bastará con escribir una frase bonita para la tarjeta.
Los que practican deportes de riesgo poseen una vida llena de emoción.
Tengo que comprar una cama para mi nuevo apartamento.
En el supermercado hay que hacer una fila para comprar el pan.
Para medir el espacio es necesario tener una unidad de distancia.
La tarea consistió en crear una imagen mental de tus amigos.
Todos deberíamos evitar una muerte sin sentido.
Mi ayuntamiento quiere arreglar una zona muy descuidada.
En este momento estás viviendo una etapa fundamental para tu desarrollo.
La ciencia dice que la luna tiene una fase ideal para su estudio.
Este verano voy a alquilar una isla para pasar las vacaciones.
Mi primo quiere tripular una nave espacial con destino a otros planetas.
El exceso de burocracia produce una ciudad mal organizada.
Por la mañana preparé una clase muy interesante.
La peste negra desencadenó una época muy oscura.
El fin no justifica los medios egoístas y abusivos.
Hubo un gran conflicto entre los actores de la obra de teatro.
Esta pintura representa el conflicto entre los toros y los humanos.
Un prisma puede dividir los rayos solares en sus componentes.
El contador presentó los costes de producción de la empresa.
Esta mañana mi hijo arrojó los restos de comida a la basura.
Debemos de anotar los gastos que hemos hecho en el viaje.
Siempre hay que leer los efectos secundarios de los medicamentos.
El cementerio es donde los muertos están enterrados.
Este hospital contrató los médicos más cualificados del país.
El gobierno eliminará los coches que funcionan con petróleo.
Hay zonas especiales para los perros de los clientes.
Me aconsejaron que recuerde los éxitos y que olvide los fracasos.
Nadie puede romper los lazos de la amistad.
Tenemos que registrar los pagos de la empresa.
En el segundo tiempo ocurrieron los minutos más emocionantes del partido.
Hay que obstaculizar los planes del enemigo cuanto antes.
La bandera de mi país contiene los colores de la libertad.
Es bueno resolver los asuntos que hemos dejado pendientes.
Este trabajo me está dando los años más valiosos de mi vida.
Hay que cuestionar el rol que tienen los ricos en la sociedad.
Las revoluciones produjeron los daños más severos a la dictadura.
Los astrónomos estudian los cuerpos celestes del sistema solar.
Hoy mi hija aprendió a distinguir los números pares de los impares.
Ayer me explicaron los motivos del porqué no me contrataron.
Esta tarde voy a pintar los muros de mi cuarto.
Hice una fiesta para los vecinos del barrio al ganar el campeonato.
Esta empresa construye los barcos que navegan por el área.
El edificio se ha derrumbado desde una altura muy considerable.
En el verano organizaré una boda para un amigo de la escuela.
El alcalde restauró una aldea afectada por la corrupción.
El peluquero afeitó una barba bien grande.
Estas vacas producen una carne de primera calidad.
El alcalde construyó una calle bien amplia.
El colegio va a implementar una medida preventiva mañana.
Mi abuelo reunió los fondos suficientes para abrir su negocio.
Mi hermana organizó una fiesta para los niños del barrio.
Tengo que esforzarme los meses que quedan de este proyecto.
Hoy he estado analizando los datos producidos por este laboratorio.
El presidente usó los métodos más prudentes para controlar las protestas.
Durante el invierno los días me parecen más cortos.
Me van a operar los labios y la nariz.
Este paciente presenta una edad muy avanzada.
Mi jefe determinó una hora de llegada para todos.
Mi jefe me regaló una caja llena de chocolates.
Hoy necesito terminar una tarea antes de medianoche.
El arquitecto necesita una semana para terminar el trabajo.
El ajedrez es muy popular entre los rusos y los americanos.
Hubo una guerra entre los estados del sur y del norte.
Hay que olvidar los tiempos malos y desagradables.
Mi abuelo se rio porque los hijos de mi tío le hicieron una broma.
Los científicos expanden los límites del conocimiento humano.
Quisiera entender mejor los bienes raíces y la bolsa de valores.
Es común que los psicólogos interpreten los sueños de uno.
Siempre hubo tensión entre los mundos de la emoción y la razón.
Mi padre me dijo que hay que evitar los lugares oscuros de la ciudad.
El profesor dijo que hay que escoger los grupos para el trabajo.
La prensa debe de ayudar a equilibrar los poderes del estado.
Hay que conservar los amigos que te valoran.
En todas las elecciones se necesita contar los votos de los ciudadanos.
El biólogo comparó los ojos de los humanos y de las ratas.
Algún día quisiera visitar los pueblos de mi país.
Es importante escribir usando los puntos y las comas de manera correcta.
Hoy he visitado los sitios turísticos principales.
El profesor nos pidió memorizar los autores más importantes.
Mi padre me contó historias sobre los indios de esa zona.
He estado meditando sobre los pasos que he tomado.
La vida consiste en escoger los retos que uno mismo se impone.
Me aburre memorizar los reyes ingleses de la Edad Media.
Este libro relata poemas sobre los dioses y sus hazañas.
El rey hizo construir una torre para la biblioteca.
Hoy voy a recibir una visita muy especial.
Esta tarde nos dimos una vuelta a la manzana.
El psicólogo dijo que la amistad implica una unión fuerte entre personas.
Felizmente solo necesito una tarde para terminar mi trabajo.
Espero que tengas una mañana muy linda.
Hoy me regalaron una cámara con mucha resolución.
Mi sobrino posee una mente realmente brillante.
Me gustaría tener una hija y un hijo.
A mi hermano le gusta una niña de su clase.
El buen samaritano realizó una acción bondadosa.
Ayer se me ocurrió una idea que cambiará el mundo.
Me urge encontrar una salida de esta situación.
La próxima semana cocinaré una comida típica de mi pueblo.
La entrada parecía una puerta de una iglesia.
Lavarse los dientes todos los días produce una boca saludable y limpia.
Mi amigo escribió una carta a su madre.
Hay que tener cuidado con decir una verdad que incomode a los demás.
Esta mañana preparé una masa deliciosa para la empanada.
Mi sobrina siempre hace una línea bajo los títulos de los temas.
Mi abuelo evitó una caída que pudo ser peligrosa.
Todos los héroes llevan una capa imponente y majestuosa.
El banco me ofreció una tasa de interés muy baja.
El gobierno recuperó los dólares que fueron robados.
Hice un banquete para los padres de los alumnos.
Mi esposa cocinó galletas para los jefes de la empresa.
Este mercado tiene los precios más baratos de la ciudad.
Hay que pesar los kilos de patatas que tenemos.
El detective consiguió los nombres de los principales sospechosos.
El director eligió los equipos que participarán en el torneo.
Hoy me golpeé fuertemente los dedos del pie con la pata de una silla.
El emperador poseía una fama poco favorable.
Mi abuelo me contó una historia sobre una chica que conoció en su juventud.
Desde la montaña se obtiene una vista realmente hermosa.
Ayer me atendió una mujer con muy buenos modales.
Él ha diseñado una agenda para su prima.
El abogado comentó que está teniendo los casos judiciales más difíciles.
El profesor exigió que copiemos los textos a mano.
Mi padre me compró los juegos que hay en mi habitación.
Mi madre me pidió limpiar los pares de zapatillas que yo uso.
En la tumba hay escondida una cruz debajo del ataúd.
Hoy me compré una camisa muy bonita.
Mañana voy a agendar una cena con mi novio.
Hay que escalar una cima muy alta.
Mi amigo me pidió que le brinde una ayuda a su madre.
El ingeniero dijo que el edificio presenta una base muy inestable.
Hay que promover los deseos de superación en la sociedad.
La filosofía clásica se enfocó en estudiar los fines últimos de las cosas.
Tengo que escribir sobre los modos diferentes de operar.
Los delincuentes robaron los objetos más valiosos de mi tío.
Hay que planificar los viajes que haremos en todo el año.
Hay que aceptar los cambios impredecibles con una sonrisa.
Voy a estudiar los temas de la asignatura.
Hay que medir los metros que hay entre estas dos columnas.
Tengo que saber los tipos de procesadores que son compatibles.
La nevera disminuye los grados de temperatura de los alimentos.
Es importante aprender los valores morales y éticos.
Hay que sembrar los campos de trigo en pimavera.
En mi universidad estudiamos los seres vivos y sus ambientes.
Hoy tengo que vaciar los discos duros que están en el trabajo.
He trabajado para los hombres más poderosos del planeta.
El detective explicó los cargos que tenía el acusado.
El sociólogo estudió los países del primer mundo.
No hay que olvidarse de lavarse los pies cuando uno se ducha.
Por la noche compraré los vinos para la fiesta.
Las técnicas de respiración ayudan a calmar los ataques de pánico.
Los profesores son tan desorganizados como los alumnos en este colegio.
Debes agregar a tu currículum los títulos que hayas obtenido.
El programador ajustó los niveles de dificultad del videojuego.
En la universidad estudiaré los siglos pasados del continente.
Debe de haber una manera de llegar a casa.
Estoy armando este regalo para una doña muy amable.
En la fiesta me senté sobre una mesa llena de comida.
Al artista le está costando esculpir una cara que se parezca a la de su madre.
El país se prepara para una guerra informática internacional.
En el grado desarrollé una teoría sobre por qué tememos a las serpientes.
Por la tarde he recogido una piedra del río muy bonita.
Esta crema ayuda a mantener una piel muy suave.
Mi mejor amiga me contó que mañana será una noche sin igual.
Este plato tradicional contiene una cola de caballo.
Mi tío escuchaba una banda de rock muy famosa en su juventud.
El alcalde gobierna una región muy bella.
El alcalde hizo construir una cárcel en mi ciudad.
Ayer por la noche tuvimos una caza muy exitosa.
Mi primo quiere aprender una lengua que no venga del latín.
Solo me hace falta una pieza para completar la colección.
Esta playa contiene una arena muy fina.
Me ofrecieron en el restaurante una salsa de tomate muy rica.
El artista escribió una letra preciosa para la cancion.
Necesitas máquinas para producir una bolsa resistente y biodegradable.
El nacimiento de mi hijo será una fecha que nunca olvidaré.
Hoy hemos tenido una suerte descomunal al poder coger ese tren.
En el elenco destaca una actriz muy talentosa.
